# Prolonged glucosuria and relapse of diabetic ketoacidosis related to SGLT2‐inhibitor therapy

**DOI:** 10.1002/edm2.117

**Published:** 2020-02-29

**Authors:** Gregory P. Westcott, Alissa R. Segal, Joanna Mitri, Florence M. Brown

**Affiliations:** ^1^ Joslin Diabetes Center Boston Massachusetts; ^2^ Beth Israel Deaconess Medical Center Boston Massachusetts; ^3^ MCPHS University Boston Massachusetts

**Keywords:** diabetic ketoacidosis, inpatient diabetes management, prolonged glucosuria, SGLT2 inhibitors

## Abstract

SGLT2 inhibitors (SGLT2i) are glucose‐lowering medications which increase the renal threshold for glucose reabsorption and promote glucosuria. Treatment with these agents raises serum ketone levels, and cases of diabetic ketoacidosis (DKA) during therapy have been reported. The duration of glucosuria and inpatient course of SGLT2i‐related DKA, however, is not well‐characterized. We report 11 inpatient cases of SGLT2i‐related DKA, including a subset of patients who experienced prolonged glucosuria and relapse of DKA during their hospitalization.

## Graphical abstract

SGLT2 inhibitors (SGLT2i) are known to increase serum ketones and predispose patients to diabetic ketoacidosis (DKA). The duration of glucosuria after stopping the medication is unknown, and the impact of SGLT2i use on the inpatient course of DKA is not well‐characterized. We identified 11 cases of SGLT2i‐related DKA, a subset of which suggest prolonged glucosuria after discontinuation of the medication.

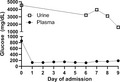

To the Editor

Sodium‐glucose cotransporter‐2 inhibitors (SGLT2i) have become increasingly popular glucose‐lowering agents, and interest in this class has broadened due to demonstration of cardioprotection in patients with established cardiovascular disease and slowing the progression of renal dysfunction.^1^ There is also interest in extending this therapy to patients without diabetes, as it appears to lower the risk of heart failure or cardiovascular death, even in patients with heart failure in the absence of diabetes.^2^ SGLT2i increase serum ketones, and a recognized adverse effect of SGLT2i therapy is diabetic ketoacidosis (DKA). While the incidence and risk factors for developing DKA on SGLT2i have been studied,^3^ and recognition and prevention of DKA have become part of society and consensus guidelines,^4^, ^5^ it has been assumed that DKA in this setting mimics classical DKA typically seen in type 1 diabetes. However, there are no detailed studies of the inpatient course of patients who develop this adverse medication effect. Therefore, we performed a systematic medical records search at a large academic medical centre in Boston, MA to identify common patterns and inpatient course for cases with DKA associated with SGLT2i.

The Beth Israel Deaconess Medical Center (BIDMC) electronic medical record was systematically queried to identify cases of DKA in patients prescribed SGLT2i. The BIDMC Institutional Review Board exempted this protocol from review. Inclusion criteria were as follows: age 18 or older, admitted between 1 January 2013 and 16 May 2019, prescribed any SGLT2i at any point, and diagnosed with DKA, either by ICD codes or using serum bicarbonate under 20 mEq/L. The records were individually reviewed by a physician to confirm that use of a SGLT2i occurred prior to the admission for DKA. DKA was defined using the American Diabetes Association definition (pH ≤ 7.30, serum bicarbonate ≤18, and positive urine ketones). Venous pH is reported, as arterial pH is not routinely obtained at this institution to diagnose DKA. Serum ketones were not available for most cases. The duration of intravenous insulin infusion, persistence of glucosuria and time to close anion gap (5), defined as time elapsed from the first available serum chemistry to the chemistry measuring an AG ≤ 16, were extracted from medical records.

A total of 273 unique patient records were identified and reviewed for inclusion. Reasons for exclusion included: no inpatient or emergency room admission for DKA (249 cases) and not on SGLT2i prior to admission (13 cases). The remaining 11 cases were analysed for this study.

Data for the 11 cases are summarized in Table 1. Patients with type 2 diabetes tended to be older and have more comorbidities than those with type 1. Among all patients, the average time to resolve DKA as defined by closure of the AG was 37.8 hours, ICU stay averaged 4.5 days, and total length of stay was 9.5 days. Our investigation reveals a subset of six patients who had prolonged ICU courses of four or more days, were on an IV insulin infusion for over 40 hours, had glucosuria for at least 3 days following admission, and in three cases, had relapse of DKA while admitted. Glucosuria persisted for up to 10 days after discontinuing the SGLT2i despite adequate glycemic control in most cases, suggesting that glucosuria is evidence of continued drug action. For one case (#5), urine glucose concentration was quantified during hospitalization (see Figure 1) and demonstrated significant elevation of urine glucose for several days after discontinuation of the drug despite normal plasma glucose levels. Of note, two cases (#7 and #8) had recently initiated a ketogenic diet.

**Table 1:** Clinical data for patients admitted with DKA in the setting of SGLT2 inhibitor use

#	DM type	SGLT2i and dose	Other admit DM meds	Age	Sex	A1c	Comorb	Admit diag.	Hosp. stay (days)	Initial glucose (mg/dL)	Initial HCO3 (mEq/L)	Initial pH (ven.)	Urine ket. (mg/dl)	Time to close AG (hours)	Duration of insulin drip (hours)	ICU stay (days)	Relapse of DKA*	Duration of glucosu. (days)	Serum glucose at latest recorded glucosu. (mg/dL)
1	1	Cana 100 mg daily	Insulin	22	F	10.1	None	DKA	3	241	3	6.89	150	24.5	22.5	3	No	ND	NA
2	1	Empa 10 mg daily	Insulin	41	F	8.8	None	DKA	4	203	12	7.27	150	23	41.5	4	Yes	> 3	113
Type 1 diabetes average				31.5	‐	9.5	‐	‐	3.5	222.0	7.5	7.08	‐	23.8	32.0	3.5	‐	‐	‐
3	2	Cana 300 mg daily	MF, Lira	41	M	7.5	HTN, HLD, OSA, obesity	DKA	5	198	5	7.03	150	99	64	4	No	>3	128
4	2	Empa 25 mg daily	MF	51	M	9.6	HTN, HLD, NASH, gastrop., HIV, obesity	Gast. outlet obs.	12	196	18	7.24	150	33.5	132	11	Yes x2	10	189
5	2	Cana 300 mg daily	MF	53	M	8.5	HTN, HLD, SAH/TBI	DKA	15	857	10	7.21	80	48	113	6	No	>9	190
6	2	Empa 25 mg daily	Dula, Insulin	56	F	10.6	CAD, HTN HLD, CKD, obesity, OSA	MRSA bact., DKA	27	282	14	7.26	80	7	28	1	No	ND	NA
7	2	Empa 25 mg daily	MF, Dula	56	M	7.3	CAD, HTN, HLD	CAD, DKA	8	127	11	7.21	150	26	31	1	No	ND	NA
8	2	Empa 25 mg daily	MF, Glip	62	M	10.2	HTN, HLD	DKA	4	472	6	7.18	80	23.5	30.5	3	No	ND	NA
9	2	Cana‐MF 150‐500 mg BID	Insulin	70	M	ND	CAD, HTN, HLD	DKA	11	547	8	7.11	40	59.5	123	8	Yes	>5	89
10	2	Empa 25 mg daily	MF, Glip	85	M	14.7	HTN, HLD	DKA, lung cancer	9	353	3	6.97	150	24.5	48	4	No	>4	313
Type 2 diabetes average				59.3	‐	9.8	‐	‐	11.4	379.0	9.4	7.15	‐	40.1	71.2	4.8	‐	‐	‐
11	3c	Cana 300 mg daily	MF	44	F	13.2	HTN, chronic panc.	DKA	6	503	9	7.24	80	47	48	4	No	ND	NA
All patients average				52.8	‐	10.1	‐	‐	9.5	361.7	9.0	7.15	‐	37.8	62.0	4.5	‐	‐	‐

Abbreviations: AG, anion gap; CAD, coronary artery disease; cana, canagliflozin; CKD, chronic kidney disease; dula, dulaglutide; empa, empagliflozin; gastrop, gastroparesis; glip, glipizide; glucose, glucosuria; HIV, human immunodeficiency virus; HLD, hyperlipidemia; HTN, hypertension; ket, ketones; lira, liraglutide; MF, metformin; NA, not applicable; NASH, nonalcoholic steatohepatitis; ND, no data; OSA, obstructive sleep apnoea; SAH, subarachnoid haemorrhage; TBI, traumatic brain injury; ven, venous.

* Reopening of AG requiring reinitiating IV insulin drip within the same hospitalization.

**Figure 1:**
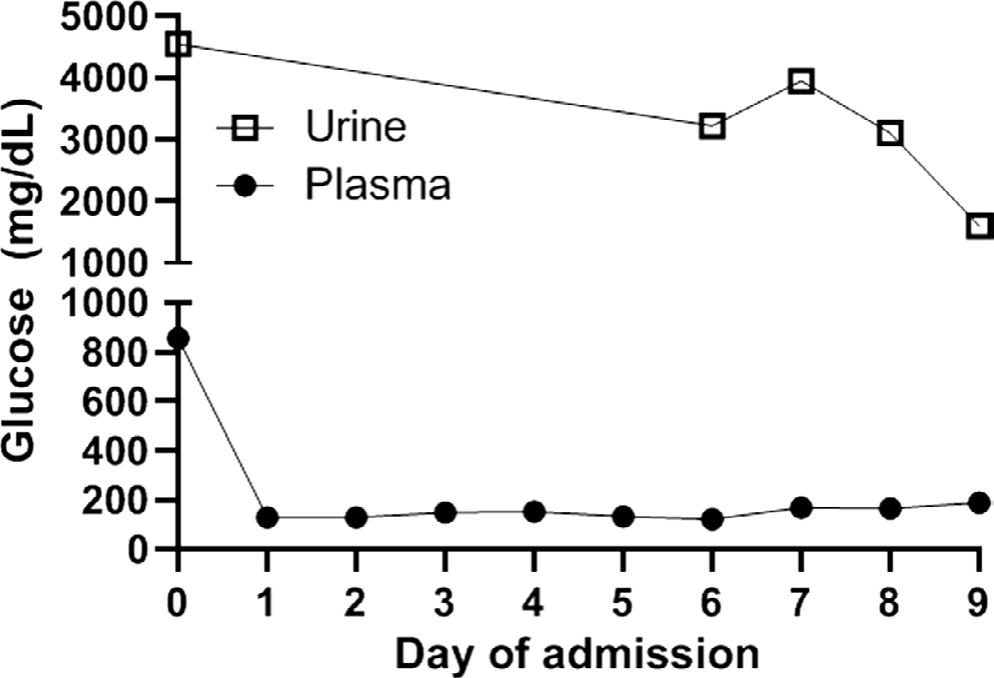
Urine and plasma glucose concentration for patient 5

Our study suggests not only that the time to close the anion gap may be longer in patients with SGLT2i‐related DKA, but also that even once the gap has closed, inadequate insulin or carbohydrate delivery can result in recurrent ketosis and risk of DKA relapse due to the ongoing glucosuric effects of the medication. Treatment of DKA relies principally on volume resuscitation and adequate insulin and carbohydrates to suppress ketosis. In classical DKA in patients with type 1 diabetes, this is often accomplished relatively quickly; multiple prior studies have indicated that median time to resolution of classical DKA is approximately 11 hours and total hospital length of stay is 3 days, though patients with classical DKA tend to be younger and with fewer comorbidities compared with those on SGLT2i.^6^, ^7^

This study is the first detailed analysis of the inpatient course of all SGLT2i‐related DKA cases within a single centre and including an evaluation of glucosuria. Prior investigations have focused primarily on defining the risk of developing DKA with SGLT2i use in large populations, indicating an increased risk in some^8^ but not all^9^, ^10^ analyses. Two more recent studies designed to identify risk factors for developing SGLT2i‐associated DKA also reported hospital stay characteristics,^3^, ^11^ though the focus was on precipitating events which lead to DKA rather than inpatient course, and duration of glucosuria was not reported. Case reports have suggested that despite half‐lives of 10‐19 hours (depending on the agent), the glucosuric effects of SGLT2i can persist up to 9 days.^12^ In our cohort of 11 patients, six had elevated urinary glucose concentrations for 3‐10 days despite cessation of the medication upon admission, and three of those had DKA relapse during their admission.

Our study suggests that patients with type 2 diabetes hospitalized with SGLT2i‐related DKA are likely older and have more comorbidities than those with type 1 diabetes, and required a longer period of time on IV insulin and in the hospital. Age and comorbidities certainly may have played a role in their clinical course, and given new SGLT2i indications for prevention of adverse cardiovascular events and progression of renal failure, increasing numbers of chronically ill patients may be started on this therapy. In cases of DKA, particularly when clinical circumstances may require prolonged fasting or oral intake is not adequate due to concomitant medical conditions or treatments, sequential urine glucose measurements may be particularly helpful to assess whether continuous intravenous insulin and dextrose should be extended. In situations where IV insulin is not readily available, frequent scheduled dosing of subcutaneous rapid‐acting insulin every 2‐4 hours with monitoring of blood glucose may be a reasonable alternative.

It is essential that inpatient therapy of SGLT2i‐related DKA focuses on providing adequate insulin and dextrose independent of serum glucose concentrations given ongoing glucosuria despite resolution of metabolic acidosis. Monitoring urine glucose concentration may also assist in the risk assessment for DKA relapse in this setting. A larger, prospective multicentre study comparing patients with DKA on SGLT2i to a control group of DKA and no SGLT2i that closely monitors urine glucose concentrations and other relevant clinical factors is necessary to define recommended monitoring and management strategies. Guidelines for treatment of SGLT2i‐related DKA should recognize that patient characteristics and course may differ from that of classical DKA and require vigilance to prevent relapse and extended hospitalization.

## CONFLICT OF INTEREST

The authors have no relevant conflicts of interest to disclose.

## AUTHOR CONTRIBUTIONS

GPW collected and analysed the data and drafted the manuscript. ARS and JM contributed to study design and reviewed/edited the manuscript. FMB conceived of study design, contributed to the data analysis and reviewed/edited the manuscript.

## Data Availability

The data that support the findings of this study are available from the corresponding author upon reasonable request.
